# Anæsthetics

**Published:** 1905-02-25

**Authors:** 


### ANÆSTHETICS.

**Chloroform.** — The committee of the British Medical Association report a series of experiments by Sherrington and Sowton[1] on the effect of chloroform on the isolated mammalian heart. Using cat's blood, they perfused an isolated cat's heart and special means were devised to prevent sedimentation of the corpuscles. Under these conditions the heart-beat was properly maintained. Blood containing known percentages of chloroform was perfused through the coronary vessels; experiments were made with practically undiluted blood, with blood diluted with serum, with blood diluted with physiological salt solution, and with Locke's salt solution with and without added dextrose. They found that chloroform dissolved in blood did not depress the heart to the same extent as when dissolved in salt solution, a result that they attributed partly to the better nutrition of a heart perfused with blood, but mostly to the greater tension of chloroform when dissolved in saline solution. They also note that in percentages of chloroform below a certain strength the heart recovers completely from the depressant action of the drug as soon as the cause is removed, though with higher percentages permanent damage occurs. Martin[2] has called attention to Embley's work, in which the chloroform mixture supplied to the isolated heart and lungs in 0·8 per cent. strength caused stoppage of the heart in 16 minutes, and in 1·2 per cent. strength in nine minutes. In the living animal five or six hours would be necessary, owing to the absorption of chloroform by the entire blood and tissues. Buxton[3] considers that this experiment shows that high percentages of chloroform alone are dangerous, and that accidents occurring with low percentages are due to the operator beginning his incision before anæsthesia is complete. The effect of Byles, Harcourt, and Horsley's[4] experiments on the estimation of chloroform dissolved in blood is to show that a considerable proportion is retained; sometimes as much as 20 per cent. It was then found that the retention of this chloroform depended on the blood corpuscles, and was not found when the blood was previously laked. A dog anæsthetised by Harcourt's inhaler was found under complete anæsthesia, and immediately after death, to contain in his blood percentages of chloroform closely corresponding to those given by earlier observers. Moore and Roaf[5] have shown that chloroform, under adequate pressure or concentration, forms loose compounds with the proteids of the blood and tissues (1) by the greater solubility of chloroform in blood or serum, and (2) by the fact that at equal concentrations the pressure is much lower in blood or serum than in water or saline solution. They think the anæsthetic effects are due to its action on protoplasm, and not on fat or lecithin. Edie and Moore[6] found that chloroform produced a precipitate insoluble in water, with solutions of hæmoglobin and serum. From this precipitate a gas, presumably chloroform, could be recovered. With carbon-monoxide hæmoglobin much more chloroform was necessary to produce a precipitate.

**Inhalers.**—The form of inhaler designed by Harcourt has been further improved in detail by him.[7] The conical bottle holding the chloroform has been accurately graduated, so that the increased surface of chloroform, as the bottle becomes empty, compensates for the smaller depth of the liquid. The variation in percentage of chloroform inhaled with normal respiration only varies by ·04 with normal and ·36 with shallow respiration. The deeper the respiration the lower the percentage of chloroform inhaled, though the absolute amount is higher. With normal respiration, the temperature being kept within the proper limits and the bottle not shaken, the amount of chloroform delivered by the apparatus will be about constant at 2 per cent. Horsley[8] confirms the advantages of this inhaler and notes that in children 1·5 per cent. vapour is enough to produce anæsthesia, and that after 20 minutes the percentage in all cases falls enormously. McCardie[9] finds the lid reflex abolished or much dulled after 16½ minutes, using 1·5 per cent. chloroform in Harcourt's inhaler. He thinks dosage can be accurately regulated by its means, that quiet induction, light anæsthesia combined with efficiency, perfect control, and quick recovery are all secured by its use. Crouch[10] considers that, shaking the apparatus being avoided, anæsthesia is usually induced in 20 minutes. With shallow respiration, however, the patient goes under quickly and there is grave danger of an overdose. Owing to the difficulty of avoiding shaking he considers the apparatus useless if not dangerous. Lilly[11] reports that even slight shaking raises the percentage of chloroform delivered through Harcourt's inhaler to 2·73, while a stronger shake raises it to 5, the index remaining at 2 per cent. He therefore considers the instrument fallacious. Waller and Wells[12] consider as the result of experiments the Harcourt apparatus can deliver known and easily regulated quantities of chloroform under laboratory conditions, and admit that its portability and cheapness are in its favour. They think, however, that for clinical purposes it is unsafe, as jolting of the bottle may raise the percentage of vapour to 5 per cent, and with a restless patient it is difficult to keep the face-piece accurately adjusted and the apparatus still. It is also based on a false principle, as the amount of chloroform delivered is regulated by the patient's respirations which may vary enough to cause a considerable overdose. Du Bois' apparatus is considered safe and trustworthy by Chapman[13], who found anæsthesia was induced in about eight minutes on the average. A strength of 2 per cent. vapour could not be exceeded unless the instrument was shaken, and the percentage was uninfluenced by the depth of the patient's respiration. Horsley[14] disapproves of it on account of its weight (half a hundredweight) and the principle on which it is constructed ("blow through"). Waller and Wells[15] find that it satisfies them under laboratory conditions; and while admitting that its weight and cost (£20) are against its clinical use maintain that these should be secondary considerations where safety and efficiency are concerned. The apparatus puts no undue strain on the attention of the anæsthetist, and the worst that a clumsy manipulator can do is to damage the instrument. Shaking can be avoided by placing it on a firm three-legged stool. Waller and Wells have also experimented with Junker's inhaler, and find that it is difficult to manipulate so as to secure a known percentage of vapour, the actual amount delivered being considerably in excess of the nominal.

[1] Brit. Med. Jour., July 23, 1904, p. 162. [2] Lancet, Aug. 20, 1904, p. 539. [3] Ibid. [4] Brit. Med. Jour., July 23, 1904, p. 169. [5] Lancet, Aug. 20, 1904, p. 538. [6] Ibid. [7] Brit. Med. Jour., July 23, 1904, p. 169. [8] Lancet, Aug 20, 1904, p. 538. [9] Ibid., p. 539. [10] Ibid. [11] Ibid. [12] Ibid., July 9, 1904, p. 77. [13] Ibid., Aug. 20, 1904, p. 539. [14] Ibid., p. 538. [15] Loc. cit.

(*To be continued.*)

